# Cohort Profile Update: Southall and Brent Revisited (SABRE) study: a UK population-based comparison of cardiovascular disease and diabetes in people of European, South Asian and African Caribbean heritage

**DOI:** 10.1093/ije/dyaa135

**Published:** 2020-10-13

**Authors:** Siana Jones, Therese Tillin, Chloe Park, Suzanne Williams, Alicja Rapala, Lamia Al Saikhan, Sophie V Eastwood, Marcus Richards, Alun D Hughes, Nishi Chaturvedi

**Affiliations:** 1 MRC Unit for Lifelong Health & Ageing at UCL, Department of Population Science & Experimental Medicine, Institute for Cardiovascular Science, UCL, London, UK; 2 Department of Cardiac Technology, College of Applied Medical Sciences, Imam Abdulrahman Bin Faisal University, Dammam, Kingdom of Saudi Arabia

## The original cohort

SABRE (Southall and Brent Revisited) is a population-based cohort study consisting of White British people and first-generation migrants of South Asian or African Caribbean heritage recruited from West London. Baseline (1988–91, *n* = 4972) and 20-year follow-up (2008–13, *n* = 1438) data collections have been described previously.^1^ Baseline initially comprised two cross-sectional studies, conducted by the same team to identical protocols and based in the London boroughs of Southall (I) and Brent (II). The studies investigated ethnic disparities in prevalence of type-2 diabetes mellitus (T2DM) and cardiovascular disease (CVD) between these three ethnic groups. Participants (80% male by design) were recruited from either their workplaces (20%) or randomly selected from general practice registers (80%).

## What is the reason for the new data collection and focus?

SABRE participants are now entering old age (mean±SD age of clinic attenders 2014–2018: 72 ± 7years). Although physical, cardiac and cognitive function are central to healthy ageing, data describing ethnic and gender differences in functional capacity in older adults are sparse and contradictory. The elevated prevalence of T2DM and susceptibility to its detrimental sequelae among ethnic minority groups remain incompletely understood. A new focus at the 25–30 year SABRE follow-up visit (2014–18) is on ethnic and sex differences in physiological and cognitive functional capacity—in association with clinical and sub-clinical cardiometabolic disease. The detailed phenotyping in SABRE provides a unique opportunity to investigate cardio-metabolic health in older age, in addition to the functional consequences of cardio-metabolic dysfunction from middle- to old-age. This will further the aetiological understanding of heart failure, exercise intolerance and cognitive decline. Advances in genetic, epigenetic and metabolomics analyses will also enhance understanding of the mechanisms underlying ethnic differences in cardiometabolic disease prevalence. This will ultimately lead to improved preventive strategies and treatments at different stages of life.

To address previous limitations of the SABRE study, in the current sweep we recruited partners of index participants with the objective of recruiting additional women and a new intake of African Caribbeans, who were, by design, recruited in lower numbers at baseline. The objective of this was to increase the power of cross-sectional analyses investigating ethnic and sex differences.

## What are the new areas of research?

The key new area of research is understanding ethnic differences in functional outcomes central to healthy aging. We aim to identify risk factors, largely from middle age, that are most strongly associated with functional outcomes in older adults measured at this recent data collection. Specific research questions for the 25–30 year follow-up include the following.

- What are the risks of, and ethnic differences in, cardiac, cognitive and physical dysfunction in older age?
- How have these progressed over 5 years [comparison with visit 2 (2008–11)] in each ethnic group?
- Which risk factors measured in mid-life and in early old age are most strongly associated with these current functional measures and with 5 year changes? Can these account for ethnic differences in function?
- How large are gender differences in current disorders of cardiac, physical and cognitive function and in their associations with current risk factors?
- Do ethnic differences in prevalence of cardiometabolic disease persist into older age?
- Which risk factors or risk factor profiles measured in mid-life and early old age are most strongly associated with incident cardiometabolic disease and which best explain ethnic differences in incidence?

## Who is in the cohort?

Table 1 provides the number of participants who attended clinic at each round of data collection. At visit 3, partners of index participants are included in the total number provided in Table 1; five partners did not self-identify as South Asian, African Caribbean or European and are identified in the category ‘other’ ethnicity. Recruitment of additional African Caribbeans did not make up for the loss of African Caribbean women from visit 2 to visit 3.

**Table 1:** Number of participants who attended each clinic visit completed the questionnaire and consented to data linkage^a^

*n* (%) or range	Original Southall and Brent studies	SABRE follow-up visit 2	SABRE follow-up visit 3
Clinic attendance			
Data collection period	1988–91	2008–11	2014–18
Age range (years)	40–69	57–90	Index participants: 65–90 New participants: 37–90
Number invited to clinic (index)	7942	3433 (address traced survivors)	1412 (address traced surviving V2 participants) All index invites included a partner invite New ACs recruited from community outreach
Total response *n* (% of those invited)	4972 (63%)	Attended clinic: *n* = 1438 (42%) Home visit: *n* = 29 (1%) Refused: 715 (21%) No response: 306 (9%) Away/unwell/other non-participation: 284 (8%)	Attended clinic: *n* = 980 (634 index, 44% of V2 attenders)^b^ Home visit: *n* = 20 (1%) Response to index participant letters^b^: refused: 246 (17%); no response: 293 (20%); away/unwell/other non-participation: 219 (15%)
Participants *n* (% of total attendance)	All index	All index	Index: 654 (65%) New partners: 249 (25%) New AC: 97 (10%)
Ethnicity of attendees *n* (men/women)	E: 1787/559 SA: 1420/291 AC: 453/348 (Other: 114/0)	E: 530/154 SA: 444/78 AC: 118/114 Other: 0	E: 164/273 SA: 134/194 AC: 146/84 Other: 3/2
Questionnaires and data linkage			
Completed questionnaires only, *n*	–	661	139 Index participants 51 New participants
Legal basis for data linkage			
Section 251^c^ *n* (%)	No linkage	4534	3469 (index) Pending (new)
Consent *n* (%)	No linkage	Not requested	572 (Index) 214 (New) (494 Lost to mortality follow-up due to departure from the UK or opt-out of data linkage)
Total deaths, *n*	–	1129	1791

a AC, African Caribbean; E European; SA, South Asian.

b Partner packs were sent out with each index participant invitation and new recruitment of African Caribbean participants was carried out by clinic staff who went into local communities, it is therefore not possible to report the exact number of invitations made.

c Section 251 of the National Health Service Act 2006.

## What has been measured?

Investigations conducted at baseline and visit 2 are detailed in the original cohort profile.^1^ All investigations conducted at visit 2 were also conducted at visit 3, with the exception of: coronary calcification on computed tomography, an oral glucose tolerance test, applanation tonometry of the radial artery (Sphygmocor) and retinal photography. In addition to the repeated investigations, a list of new investigations and key measurements from each test is provided in Table 2.

**Table 2:** New investigations conducted at SABRE study follow-up visit 3 (2014–18) and their key measurements^a^

Investigation	Key measurements	Sub-sample
Physical function		
6MST	Exercise capacity Heart rate Sub-maximal and estimated maximum whole-body oxygen consumption (VO_2_) BP response	
Grip strength	Muscle strength BP response to grip (30% of max)	
Datagait test	Stride length Self-selected walking pace Walking efficiency and control	
Risk of fall ‘Quick Screen’	Visual acuity Tactile sensitivity Near tandem stand test Alternate step test Sit-to-stand test	
Cardiovascular/respiratory		
Orthostatic challenge	Change in BP from lying to standing	
Vicorder	Pulse wave velocity	
5-min resting ECG	Heart rate variability	
ASL (added to cerebral MRI protocol)	Cerebral blood flow	
Spirometry	Forced vital capacity (FVC) Forced expired volume in first second (FEV_1_)	
Body composition		
DEXA	Body composition (fat/lean mass) Bone mineral content and density (hip and lumbar spine) Aortic calcification	
Metabolic		
Liver ultrasound	Elastography Hepato–renal ratio	
Non-fasting blood sample (after early light breakfast) Saliva sample	Stored samples (whole blood, plasma, serum, PAXgene RNA, PAXgene DNA) Cortisol, amylase	
Scout AGE reader (forearm)	Advanced glycation end products (AGEs)	√
Muscle NIRS	Muscle haemodynamic response to exercise Local muscle oxygen consumption Muscle oxidative capacity	√ √
Questionnaire		
Self-report	Perceptions of physical disability and ageing. Hearing, vision, dental health. Caring responsibilities and need for care, receipt of benefit payments, healthcare resource use. Reproductive health and menopause. Childhood diet and physical activity	

a AGEs, advanced glycation end-products; ASL, arterial spin labelling; BP, blood pressure; NIRS, near-infrared spectroscopy.

Alongside the focus on physical function, the self-completion questionnaire now includes questions on perceptions of physical disability and ageing, vision, hearing and dental health, need for carer, or caring responsibilities, receipt of disability-related benefit payments, healthcare resource use and social contact. Furthermore, questions have been added on childhood diet and physical activity to improve understanding of life-course influences. Finally, to aid understanding of sex differences in cardiometabolic disorders and function, we have added questions on reproductive health and menopause to the self-completion questionnaires.

Key to the new focus of the study is the addition of physical function assessment. We used a self-paced, 6 min stepper test (6MST) to assess exercise capacity;^2^^,^^3^ this is similar to the 6 min walk test, but conducted on a stepper in a static environment, permitting more accurate assessment of haemodynamic changes during exercise (blood pressure and muscle blood flow). Skeletal muscle haemodynamics were investigated using near-infrared spectroscopy during and following exercise.^4^^,^^5^ Quick Screen tests were performed in all participants as a multifactorial falls risk assessment.^6^

### Data linkage; mortality & hospital episode statistics

Following visit 1, participants have been flagged for death and causes of death by the UK Office for National Statistics. There has been some loss to mortality flagging follow-up (*n* = 373(8%)) due to departure from the UK and withdrawal of consent or non-reply to consent requests.

Hospital Episode Statistics are provided by NHS Digital and cover inpatient admissions since 1989. Data include dates and methods of admission and discharge, International Classification of Disease diagnostic codes and operation codes (OPCS classification of interventions and procedures). Clinic-attending participants were asked to give consent for data linkage during their visit. Participants who do not attend clinic were given an opportunity, by post, to opt out of data linkage.

## Key findings and publications

### T2DM and adiposity

South Asian and African Caribbean migrants continue to experience excess incidence of T2DM into older age, such that, by the age of 80, half of all migrants have T2DM, compared with a fifth of Europeans.^7^ Mid-life insulin resistance and truncal obesity accounted for most of this excess risk in women but not in men.^7^ Whereas South Asians have more visceral fat, African Caribbeans have less, though both ethnic groups have greater truncal skinfolds than Europeans.^8^ More specifically, greater visceral and thigh subcutaneous adiposity combined with lesser thigh muscle in South Asians contributed to their elevated cardiometabolic disease risk.^9^ Longitudinal analyses confirmed that associations between truncal skinfolds (mid-life) and diabetes were stronger in South Asians and African Caribbeans than in Europeans.^8^ Not only did the ethnic minorities experience more T2DM, they also had equivalent diabetes incidence rates at substantially lower obesity levels than the conventional European cut-points.^10^

### Metabolomics

Metabolite profiling of baseline blood samples implicated branched chain and aromatic amino acids, particularly tyrosine, in the aetiology of subsequent T2DM development in South Asians.^11^ Combined with metabolomics data from two other population-based cohorts, these data also contributed to the identification of phenylalanine, monounsaturated fatty acids and polyunsaturated fatty acids as biomarkers for cardiovascular risk, substantiating the value of high-throughput metabolomics for biomarker discovery and improved risk assessment.^12^

### CVD

The SABRE study confirmed an ongoing excess coronary heart disease (CHD) incidence in South Asians, with lower incidence in African Caribbeans compared with Europeans in early old-age, and also confirmed an elevated risk of stroke in both ethnic minority groups.^13^ Measured baseline metabolic risk factors could not fully explain the ethnic group differences in CHD; however, there was a markedly stronger association between diabetes and stroke risk in both ethnic minority groups compared with Europeans with diabetes.^13^^,^^14^ Additionally, we showed that mid-life elevated mean arterial pressure was more important than pulse pressure as a risk factor for stroke in South Asians, whereas the converse was true for Europeans.^15^ Finally, age and other cardiovascular risk factors were associated with a greater degree of white matter hyperintensities on cerebral magnetic resonance imaging (MRI)—a subclinical measure of brain damage as a consequence of cardiovascular risk factors, and a potent determinant of future stroke risk in South Asians compared with Europeans.^16^

Detailed cardiovascular phenotyping in SABRE has revealed that left ventricular (LV) function and arterial stiffness (central pulse pressure/stroke volume) are more adversely affected by T2DM and hyperglycaemia in South Asians than in Europeans.^17^^,^^18^ Using 3D-echocardiography we also observed lower LV mass in South Asians compared with Europeans or African Caribbeans. Contrary to findings using conventional 2D echocardiography, 3 D-echocardiography, which makes no assumptions regarding LV geometry, showed that LV mass was similar in Europeans and African Caribbeans and that LV remodelling accounted for differences in LV structure in African Caribbeans.^19^ The role of LV remodelling in the excess of heart failure in people of African heritage merits closer investigation. Echocardiography in SABRE has provided insight into sex-differences in cardiovascular outcomes predicted by differential LV structure and function.^20^^,^^21^

In addition to these analyses of detailed clinical measurements, a combined longitudinal analysis using SABRE and the National Survey of Health and Development (NSHD) cohort (Dehbi *et al*.^22^) reported detrimental associations between air pollution (particulate measures) and CVD mortality.

### Cardiovascular risk identification tools

Tillin *et al*. identified that two widely used cardiovascular risk prediction tools (QRISK2 and Framingham) did not perform consistently well in all ethnic groups and suggested that better methods for identifying high-risk African Caribbeans and South Asian women are required.^23^ Furthermore, the association between pre-diabetes and risk of CVD differed, not only by ethnicity and type of CVD (CHD vs stroke) but also by the criteria used to establish pre-diabetes (either International Expert Committee (IEC) [Glycated haemoglobin (HbA1c) 6.0–6.5% (42–48 mmol/mol)] or American Diabetes Association (ADA) [HbA1c 5.7–6.5% (39–48 mmol/mol)] cut points.^14^

### Cognitive function and depression

LV dysfunction across all ethnicities was associated with impaired cognitive function and lower total and hippocampal brain volumes.^24^ Depressive symptoms were higher among South Asians and African Caribbeans; however, these differences were explained in South Asians mostly by reduced physical health and in African Caribbeans by socio-economic disadvantage.^25^^,^^26^

A full list of findings published in peer-reviewed Journals can be found on the study website (https://www.sabrestudy.org/? p=186).

## What are the main strengths and weaknesses?

Strengths of the SABRE study are:

- large tri-ethnic cohort with 30 years follow-up;
- availability of longitudinal data at mid-life, 20-year follow-up and 25-year follow-up;
- range and detail of phenotypic measurements;
- extensive cardiovascular assessments including advanced imaging techniques (3D and 4D echocardiography), carotid imaging, central haemodynamics and pulse wave velocity;
- extensive cognitive function tests and cerebral imaging (MRI including arterial spin labelling to measure cerebral blood flow);
- quantification of body composition (fat and lean mass, bone density and aortic calcification) by DEXA);
- metabolomics and genomic profiling;
- linkage to hospital admissions and mortality register.

The main weaknesses of the study are the lack of follow-up visits between baseline and 20-year follow-up and cohort attrition over time. We aimed to boost study participants and addressed previous limitations of the lower number of African Caribbeans and women by inviting partners of index participants and a new group of African Caribbeans to attend the 25-year study clinic.

## Can I get hold of the data? Where can I find out more?

Data sharing applications are welcome. Please contact Dr Therese Tillin with an outline of proposed analyses or query t.tillin@ucl.ac.uk.

## Funding

This work was supported at baseline by the Medical Research Council, Diabetes UK and the British Heart Foundation. At follow-up the study was funded by the Wellcome Trust [grant numbers 067100, 37055891 and 086676/7/08/Z], the British Heart Foundation [grant numbers PG/06/145, PG/08/103/26133, PG/12/29/29497 and CS/13/1/30327] and Diabetes UK [grant number 13/0004774]. The study team also acknowledges the support of the National Institute of Health Research Clinical Research Network [grant number NIHR CRN]. ADH and NC work in a unit that receives support from the UK Medical Research Council [grant number MC_UU_12019/1].
